# *catena*-Poly[[diaqua­bis(diphenyl­acetato)­zinc(II)]-μ-4,4′-bipyridine]

**DOI:** 10.1107/S1600536809004450

**Published:** 2009-02-18

**Authors:** Shan-Shan Yu, Hong Zhou, Hua Xian, Zheng-Fang Tian

**Affiliations:** a Department of Chemistry, Nanjing Xiaozhuang College, Nanjing 210017, People’s Republic of China; b College of Chemistry and Applied Chemistry, Huanggang Normal University, Huanggang 438000, People’s Republic of China

## Abstract

In the title compound, [Zn(C_14_H_11_O_2_)_2_(C_10_H_8_N_2_)(H_2_O)_2_]_*n*_, the Zn^II^ ion lies on a crystallographic inversion center and is in a slightly distorted octahedral coordination enviroment. 4,4′-Bipyridine ligands act as bridging ligands, connecting Zn^II^ ions into a chain along the *b*-axis direction. In the crystal structure, these chains are linked by inter­molecular O—H⋯O hydrogen bonds to form a two-dimensional network parallel to the *ab* plane.

## Related literature

For background information, see: Janiak (2003 ▶); Moulton & Zaworotko (2001 ▶); Brammer (2004 ▶). For the role of weak noncovalent inter­actions in crystalline architectures, see: Hosseini (2005 ▶); Nishio (2004 ▶).
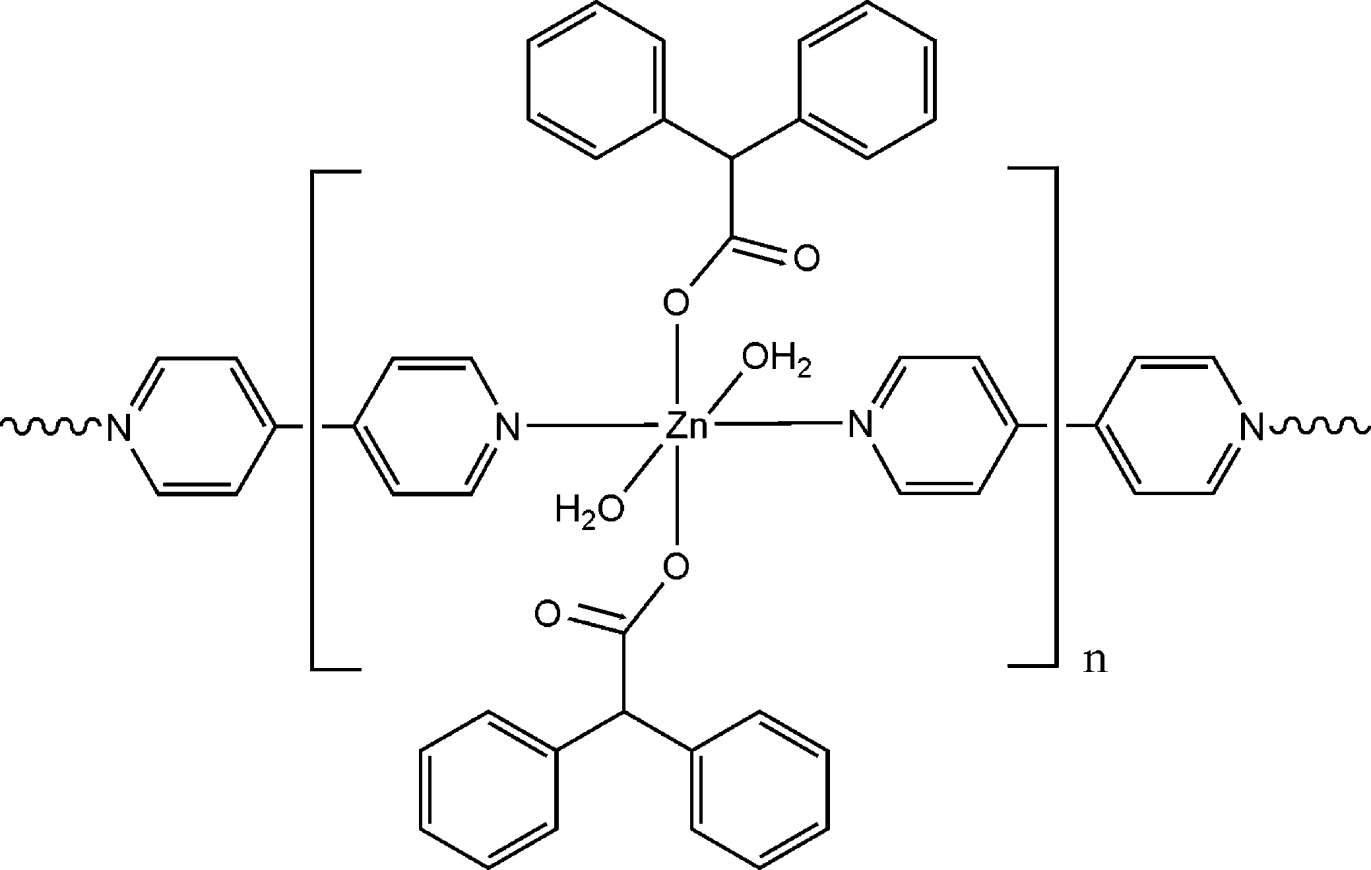


## Experimental

### 

#### Crystal data

[Zn(C_14_H_11_O_2_)_2_(C_10_H_8_N_2_)(H_2_O)_2_]
*M* *_r_* = 680.04
Triclinic, 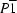

*a* = 5.7536 (13) Å
*b* = 11.882 (3) Å
*c* = 12.229 (3) Å
α = 98.522 (4)°
β = 103.273 (5)°
γ = 103.450 (4)°
*V* = 773.2 (3) Å^3^
*Z* = 1
Mo *K*α radiation
μ = 0.85 mm^−1^
*T* = 291 K
0.30 × 0.26 × 0.24 mm

#### Data collection

Bruker SMART CCD diffractometer
Absorption correction: multi-scan (*SADABS*; Bruker, 2001 ▶) *T*_min_ = 0.785, *T*_max_ = 0.823
3891 measured reflections
2679 independent reflections
2234 reflections with *I* > 2σ(*I*)
*R*_int_ = 0.022

#### Refinement

*R*[*F* ^2^ > 2σ(*F* ^2^)] = 0.061
*wR*(*F* ^2^) = 0.135
*S* = 1.02
2679 reflections
214 parameters
H-atom parameters constrained
Δρ_max_ = 0.23 e Å^−3^
Δρ_min_ = −0.22 e Å^−3^

### 

Data collection: *SMART* (Bruker, 2001 ▶); cell refinement: *SAINT* (Bruker, 2001 ▶); data reduction: *SAINT*; program(s) used to solve structure: *SHELXTL* (Sheldrick, 2008 ▶); program(s) used to refine structure: *SHELXTL*; molecular graphics: *SHELXTL*; software used to prepare material for publication: *SHELXTL*.

## Supplementary Material

Crystal structure: contains datablocks global, I. DOI: 10.1107/S1600536809004450/lh2759sup1.cif

Structure factors: contains datablocks I. DOI: 10.1107/S1600536809004450/lh2759Isup2.hkl

Additional supplementary materials:  crystallographic information; 3D view; checkCIF report

## Figures and Tables

**Table 1:** Hydrogen-bond geometry (Å, °)

*D*—H⋯*A*	*D*—H	H⋯*A*	*D*⋯*A*	*D*—H⋯*A*
O3—H3*B*⋯O2	0.96	1.82	2.618 (5)	139
O3—H3*C*⋯O1^i^	0.96	1.97	2.802 (5)	143

Symmetry code: (i) 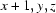.

**Fig. 1.**
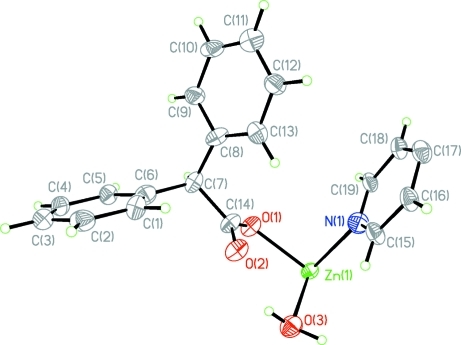
The asymmetric unit of (I), showing displacement ellipsoids at the 30% probability level.

**Fig. 2.**
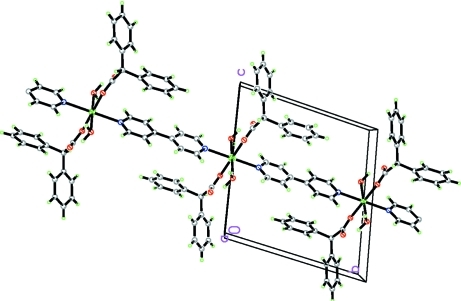
Part of the one-dimensional chain structure of (I).

## supplementary crystallographic information

### Comment

During the past decade, the design of new metal-organic supramolecular solids has attracted attention in the fields of coordination chemistry and crystal engineering, for the sake of developing desired crystalline materials with potential functionality (Moulton & Zaworotko, 2001; Janiak , 2003). Furthermore, it has been realised that weak noncovalent interactions such as hydrogen bonds, aromatic stacking, and van der Waals forces (Hosseini, 2005; Nishio, 2004) are crucial in the direction of such crystalline architectures. Hitherto, a variety of organic connectors containing pyridyl and/or carboxylate groups (Brammer, 2004) have been widely used to construct metal-organic supramolecular frameworks. Herein we report the crystal structure of the title compound (1).

The asymmetric unit of (I) is illustrated in Fig. 1. The structure of (I) is a one-dimensional chain (Fig. 2), in which the Zn^II^ ions are coordinated by two O atoms from two monodentate carboxylate groups of two bis(diphenylacetato) ligands, two N atoms of two bridging 4,4'-bipyridine ligands and two O atoms from two water molecules. The Zn^II^ ion is in a slightly distorted octahedral coordination environment. In the crystal structure, these one-dimensional chains are linked via intermolecular O—H···O hydrogen bonds to form a two-dimensional network.

### Experimental

Soild ZnCl_2_(136 mg, 1 mmol), 4,4'-bipyridine (1 mmol, 0.156 g) and diphenylacetic acid (212 mg, 1 mmol) in water (8 ml) was placed in a Teflon-lined stainless-steel Parr bomb that was heated at 433 K for 48 h. Colorless block crystals were collected after the bomb was subsequently allowed to cool to room temperature.

### Refinement

The C-bound H atoms were placed to the bonded parent atoms in geometrically idealized positions (C—H = 0.93, and 0.98 Å) and refined as riding atoms, with *U*_iso_(H) = 1.2*U*_eq_(C). The O-bound H atoms were located in difference Fourier maps and refined as riding in their as-found positions but with O—H = 0.96 Å and with *U*_iso_(H) = 1.5*U*_eq_(C).

### Crystal data

**Table:**

[Zn(C_14_H_11_O_2_)_2_(C_10_H_8_N_2_)(H_2_O)_2_]	*Z* = 1
*M**_r_* = 680.04	*F*(000) = 354
Triclinic, *P*1	*D*_x_ = 1.460 Mg m^−^^3^
Hall symbol: -P 1	Mo *K*α radiation, λ = 0.71073 Å
*a* = 5.7536 (13) Å	Cell parameters from 924 reflections
*b* = 11.882 (3) Å	θ = 2.2–20.2°
*c* = 12.229 (3) Å	µ = 0.85 mm^−^^1^
α = 98.522 (4)°	*T* = 291 K
β = 103.273 (5)°	Block, colorless
γ = 103.450 (4)°	0.30 × 0.26 × 0.24 mm
*V* = 773.2 (3) Å^3^	

### Data collection

**Table:**

Bruker SMART CCD diffractometer	2679 independent reflections
Radiation source: fine-focus sealed tube	2234 reflections with *I* > 2σ(*I*)
graphite	*R*_int_ = 0.022
φ and ω scans	θ_max_ = 25.0°, θ_min_ = 1.8°
Absorption correction: multi-scan (*SADABS*; Bruker, 2001)	*h* = −6→6
*T*_min_ = 0.785, *T*_max_ = 0.823	*k* = −12→14
3891 measured reflections	*l* = −14→11

### Refinement

**Table:**

Refinement on *F*^2^	Primary atom site location: structure-invariant direct methods
Least-squares matrix: full	Secondary atom site location: difference Fourier map
*R*[*F*^2^ > 2σ(*F*^2^)] = 0.061	Hydrogen site location: inferred from neighbouring sites
*wR*(*F*^2^) = 0.135	H-atom parameters constrained
*S* = 1.02	*w* = 1/[σ^2^(*F*_o_^2^) + (0.05*P*)^2^ + 1.22*P*] where *P* = (*F*_o_^2^ + 2*F*_c_^2^)/3
2679 reflections	(Δ/σ)_max_ < 0.001
214 parameters	Δρ_max_ = 0.23 e Å^−^^3^
0 restraints	Δρ_min_ = −0.22 e Å^−^^3^

### Special details

**Table:**

Geometry. All e.s.d.'s (except the e.s.d. in the dihedral angle between two l.s. planes) are estimated using the full covariance matrix. The cell e.s.d.'s are taken into account individually in the estimation of e.s.d.'s in distances, angles and torsion angles; correlations between e.s.d.'s in cell parameters are only used when they are defined by crystal symmetry. An approximate (isotropic) treatment of cell e.s.d.'s is used for estimating e.s.d.'s involving l.s. planes.
Refinement. Refinement of *F*^2^ against ALL reflections. The weighted *R*-factor *wR* and goodness of fit *S* are based on *F*^2^, conventional *R*-factors *R* are based on *F*, with *F* set to zero for negative *F*^2^. The threshold expression of *F*^2^ > σ(*F*^2^) is used only for calculating *R*-factors(gt) *etc*. and is not relevant to the choice of reflections for refinement. *R*-factors based on *F*^2^ are statistically about twice as large as those based on *F*, and *R*- factors based on ALL data will be even larger.

### Fractional atomic coordinates and isotropic or equivalent isotropic displacement parameters (Å^2^)

**Table:**

	*x*	*y*	*z*	*U*_iso_*/*U*_eq_	
C1	0.5242 (9)	0.7816 (4)	−0.0135 (4)	0.0552 (12)	
H1	0.6390	0.7442	0.0197	0.066*	
C2	0.4992 (9)	0.7953 (4)	−0.1250 (4)	0.0575 (12)	
H2	0.5969	0.7669	−0.1672	0.069*	
C3	0.3272 (9)	0.8519 (4)	−0.1754 (4)	0.0540 (11)	
H3	0.3106	0.8614	−0.2507	0.065*	
C4	0.1819 (9)	0.8936 (4)	−0.1118 (4)	0.0592 (13)	
H4	0.0679	0.9317	−0.1441	0.071*	
C5	0.2073 (8)	0.8782 (4)	0.0000 (4)	0.0481 (11)	
H5	0.1076	0.9052	0.0418	0.058*	
C6	0.3773 (8)	0.8238 (4)	0.0501 (4)	0.0544 (12)	
C7	0.3955 (8)	0.8080 (4)	0.1714 (4)	0.0528 (12)	
H7	0.2720	0.8423	0.1963	0.063*	
C8	0.3306 (8)	0.6802 (4)	0.1832 (4)	0.0550 (12)	
C9	0.0736 (9)	0.6218 (4)	0.1441 (4)	0.0588 (13)	
H9	−0.0450	0.6615	0.1215	0.071*	
C10	0.0081 (9)	0.4974 (4)	0.1416 (4)	0.0575 (13)	
H10	−0.1592	0.4555	0.1163	0.069*	
C11	0.1787 (9)	0.4365 (5)	0.1745 (4)	0.0557 (12)	
H11	0.1303	0.3552	0.1695	0.067*	
C12	0.4204 (8)	0.4999 (4)	0.2145 (4)	0.0527 (11)	
H12	0.5387	0.4609	0.2393	0.063*	
C13	0.4981 (10)	0.6189 (4)	0.2200 (4)	0.0552 (12)	
H13	0.6662	0.6588	0.2489	0.066*	
C14	0.6538 (8)	0.8770 (4)	0.2592 (4)	0.0463 (11)	
C15	1.1601 (9)	0.7492 (4)	0.4541 (4)	0.0494 (11)	
H15	1.2639	0.7935	0.4184	0.059*	
C16	1.1620 (8)	0.6347 (4)	0.4540 (4)	0.0478 (11)	
H16	1.2728	0.6045	0.4222	0.057*	
C17	1.0046 (9)	0.5634 (4)	0.4995 (4)	0.0510 (11)	
C18	0.8504 (9)	0.6187 (4)	0.5508 (4)	0.0489 (11)	
H18	0.7424	0.5755	0.5853	0.059*	
C19	0.8580 (9)	0.7341 (4)	0.5501 (4)	0.0476 (11)	
H19	0.7515	0.7669	0.5829	0.057*	
N1	1.0118 (8)	0.8015 (3)	0.5046 (4)	0.0591 (10)	
O1	0.6471 (6)	0.9293 (3)	0.3557 (3)	0.0600 (9)	
O2	0.8431 (5)	0.8730 (3)	0.2284 (3)	0.0516 (8)	
O3	1.2454 (6)	1.0195 (3)	0.3733 (3)	0.0608 (9)	
H3B	1.1539	0.9696	0.2994	0.073*	
H3C	1.3925	0.9961	0.4024	0.073*	
Zn1	1.0000	1.0000	0.5000	0.0473 (3)	

### Atomic displacement parameters (Å^2^)

**Table:**

	*U*^11^	*U*^22^	*U*^33^	*U*^12^	*U*^13^	*U*^23^
C1	0.053 (3)	0.063 (3)	0.046 (3)	0.020 (2)	0.004 (2)	0.010 (2)
C2	0.053 (3)	0.059 (3)	0.055 (3)	0.003 (2)	0.026 (2)	0.001 (2)
C3	0.051 (3)	0.056 (3)	0.049 (3)	0.008 (2)	0.010 (2)	0.011 (2)
C4	0.053 (3)	0.051 (3)	0.065 (3)	0.011 (2)	−0.007 (2)	0.028 (2)
C5	0.047 (2)	0.046 (2)	0.055 (3)	0.024 (2)	0.008 (2)	0.012 (2)
C6	0.046 (3)	0.052 (3)	0.061 (3)	0.014 (2)	0.012 (2)	0.004 (2)
C7	0.044 (2)	0.061 (3)	0.045 (2)	0.018 (2)	0.001 (2)	0.000 (2)
C8	0.042 (2)	0.051 (3)	0.056 (3)	0.002 (2)	0.006 (2)	−0.008 (2)
C9	0.062 (3)	0.057 (3)	0.050 (3)	0.000 (2)	0.016 (2)	0.013 (2)
C10	0.053 (3)	0.059 (3)	0.048 (3)	−0.013 (2)	0.024 (2)	0.005 (2)
C11	0.060 (3)	0.063 (3)	0.048 (3)	0.018 (2)	0.028 (2)	0.001 (2)
C12	0.049 (3)	0.049 (3)	0.065 (3)	0.016 (2)	0.018 (2)	0.020 (2)
C13	0.063 (3)	0.057 (3)	0.051 (3)	0.019 (2)	0.019 (2)	0.019 (2)
C14	0.039 (2)	0.045 (2)	0.047 (2)	0.0136 (18)	0.0069 (19)	−0.0099 (19)
C15	0.061 (3)	0.047 (3)	0.058 (3)	0.030 (2)	0.027 (2)	0.026 (2)
C16	0.051 (3)	0.057 (3)	0.054 (3)	0.032 (2)	0.025 (2)	0.026 (2)
C17	0.059 (3)	0.043 (2)	0.056 (3)	0.025 (2)	0.015 (2)	0.011 (2)
C18	0.054 (3)	0.058 (3)	0.049 (2)	0.030 (2)	0.019 (2)	0.021 (2)
C19	0.056 (3)	0.041 (2)	0.050 (3)	0.023 (2)	0.010 (2)	0.017 (2)
N1	0.065 (3)	0.044 (2)	0.064 (3)	0.0170 (19)	0.011 (2)	0.0041 (19)
O1	0.0470 (18)	0.067 (2)	0.057 (2)	0.0143 (16)	0.0080 (15)	−0.0010 (16)
O2	0.0419 (17)	0.0550 (19)	0.0550 (18)	0.0178 (14)	0.0141 (14)	−0.0054 (14)
O3	0.058 (2)	0.056 (2)	0.061 (2)	0.0140 (16)	0.0108 (16)	0.0031 (16)
Zn1	0.0440 (4)	0.0420 (4)	0.0436 (4)	0.0021 (3)	0.0025 (3)	0.0020 (3)

### Geometric parameters (Å, °)

**Table:**

C1—C2	1.377 (6)	C12—H12	0.9300
C1—C6	1.398 (6)	C13—H13	0.9300
C1—H1	0.9300	C14—O2	1.240 (5)
C2—C3	1.401 (7)	C14—O1	1.263 (5)
C2—H2	0.9300	C15—C16	1.363 (6)
C3—C4	1.388 (7)	C15—N1	1.368 (6)
C3—H3	0.9300	C15—H15	0.9300
C4—C5	1.385 (6)	C16—C17	1.365 (6)
C4—H4	0.9300	C16—H16	0.9300
C5—C6	1.374 (6)	C17—C18	1.420 (6)
C5—H5	0.9300	C17—C17^i^	1.497 (8)
C6—C7	1.505 (7)	C18—C19	1.362 (6)
C7—C8	1.514 (7)	C18—H18	0.9300
C7—C14	1.572 (6)	C19—N1	1.328 (6)
C7—H7	0.9800	C19—H19	0.9300
C8—C13	1.373 (7)	N1—Zn1	2.384 (4)
C8—C9	1.413 (6)	O1—Zn1	2.250 (3)
C9—C10	1.432 (7)	O3—Zn1	2.326 (3)
C9—H9	0.9300	O3—H3B	0.9600
C10—C11	1.372 (7)	O3—H3C	0.9600
C10—H10	0.9300	Zn1—O1^ii^	2.250 (3)
C11—C12	1.354 (7)	Zn1—O3^ii^	2.326 (3)
C11—H11	0.9300	Zn1—N1^ii^	2.384 (4)
C12—C13	1.367 (6)		
			
C2—C1—C6	120.1 (5)	C8—C13—H13	119.7
C2—C1—H1	120.0	O2—C14—O1	126.4 (4)
C6—C1—H1	120.0	O2—C14—C7	117.4 (4)
C1—C2—C3	120.3 (5)	O1—C14—C7	116.2 (4)
C1—C2—H2	119.9	C16—C15—N1	122.6 (4)
C3—C2—H2	119.9	C16—C15—H15	118.7
C4—C3—C2	119.3 (4)	N1—C15—H15	118.7
C4—C3—H3	120.3	C15—C16—C17	121.4 (4)
C2—C3—H3	120.3	C15—C16—H16	119.3
C5—C4—C3	119.8 (4)	C17—C16—H16	119.3
C5—C4—H4	120.1	C16—C17—C18	115.3 (4)
C3—C4—H4	120.1	C16—C17—C17^i^	123.8 (5)
C6—C5—C4	121.1 (5)	C18—C17—C17^i^	120.9 (5)
C6—C5—H5	119.4	C19—C18—C17	121.1 (4)
C4—C5—H5	119.4	C19—C18—H18	119.4
C5—C6—C1	119.4 (5)	C17—C18—H18	119.4
C5—C6—C7	119.0 (4)	N1—C19—C18	122.5 (4)
C1—C6—C7	121.6 (4)	N1—C19—H19	118.8
C6—C7—C8	114.4 (4)	C18—C19—H19	118.8
C6—C7—C14	113.9 (4)	C19—N1—C15	117.1 (4)
C8—C7—C14	109.1 (4)	C19—N1—Zn1	120.2 (3)
C6—C7—H7	106.3	C15—N1—Zn1	122.6 (3)
C8—C7—H7	106.3	C14—O1—Zn1	119.4 (3)
C14—C7—H7	106.3	Zn1—O3—H3B	109.4
C13—C8—C9	120.2 (5)	Zn1—O3—H3C	109.2
C13—C8—C7	125.6 (4)	H3B—O3—H3C	109.5
C9—C8—C7	114.0 (4)	O1—Zn1—O1^ii^	180.000 (1)
C8—C9—C10	115.4 (5)	O1—Zn1—O3	92.45 (12)
C8—C9—H9	122.3	O1^ii^—Zn1—O3	87.55 (12)
C10—C9—H9	122.3	O1—Zn1—O3^ii^	87.55 (12)
C11—C10—C9	123.6 (5)	O1^ii^—Zn1—O3^ii^	92.45 (12)
C11—C10—H10	118.2	O3—Zn1—O3^ii^	180.000 (1)
C9—C10—H10	118.2	O1—Zn1—N1^ii^	90.93 (13)
C12—C11—C10	117.1 (5)	O1^ii^—Zn1—N1^ii^	89.07 (13)
C12—C11—H11	121.4	O3—Zn1—N1^ii^	86.86 (13)
C10—C11—H11	121.4	O3^ii^—Zn1—N1^ii^	93.14 (13)
C11—C12—C13	122.8 (5)	O1—Zn1—N1	89.07 (13)
C11—C12—H12	118.6	O1^ii^—Zn1—N1	90.93 (13)
C13—C12—H12	118.6	O3—Zn1—N1	93.14 (13)
C12—C13—C8	120.7 (5)	O3^ii^—Zn1—N1	86.86 (13)
C12—C13—H13	119.7	N1^ii^—Zn1—N1	180.000 (2)

Symmetry codes: (i) −*x*+2, −*y*+1, −*z*+1; (ii) −*x*+2, −*y*+2, −*z*+1.

### Hydrogen-bond geometry (Å, °)

**Table:**

*D*—H···*A*	*D*—H	H···*A*	*D*···*A*	*D*—H···*A*
O3—H3B···O2	0.96	1.82	2.618 (5)	139
O3—H3C···O1^iii^	0.96	1.97	2.802 (5)	143

Symmetry codes: (iii) *x*+1, *y*, *z*.
